# Brain water as a function of age and weight in normal rats

**DOI:** 10.1371/journal.pone.0249384

**Published:** 2021-09-15

**Authors:** Allan Gottschalk, Susanna Scafidi, Thomas J. K. Toung

**Affiliations:** 1 Departments of Anesthesiology and Critical Care Medicine, Johns Hopkins University, Baltimore, Maryland, United States of America; 2 Department of Neurosurgery, Johns Hopkins University, Baltimore, Maryland, United States of America; Uniformed Services University, UNITED STATES

## Abstract

Rats are frequently used for studying water content of normal and injured brain, as well as changes in response to various osmotherapeutic regimens. Magnetic resonance imaging in humans has shown that brain water content declines with age as a result of progressive myelination and other processes. The purpose of this study was to quantify changes in brain water content during rat development and aging. Brain water content was measured by standard techniques in 129 normal male Sprague-Dawley rats that ranged in age (weight) from 13 to 149 days (18 to 759 g). Overall, the results demonstrated a decrease in water content from 85.59% to 76.56% with increasing age (weight). Nonlinear allometric functions relating brain water to age and weight were determined. These findings provide age-related context for prior rat studies of brain water, emphasize the importance of using similarly aged controls in studies of brain water, and indicate that age-related changes in brain water content are not specific to humans.

## Introduction

Rats continue to be used extensively in laboratory medicine and have been the source of considerable animal data regarding water content of the normal and injured brain [1–3]. Many researchers also have used rats to examine the extent to which various osmotherapeutic regimens can reduce brain water content [2, 3]. Magnetic resonance imaging (MRI) has shown that brain water content decreases with increasing gestational age in humans and rabbits [4, 5]. This decline in water content reflects increasing myelination and other processes [6–10]. Such findings suggest that, at least in humans and rabbits, not only does brain water vary with age, but that brain water content is tightly coupled to age. The purpose of this study was to determine whether brain water content in normal rats is also dependent on age and to thereby provide important age-related context for prior and future studies of brain water content.

## Materials and methods

All animal protocols were approved by the Johns Hopkins Institutional Animal Care and Use Committee and were carried out in male Sprague-Dawley rats (Charles River Laboratories, Inc., Wilmington, DE, USA). Before being used for the experiments, animals were fed with a standard rodent diet (Teklad, Madison, WI, U.S.A.) and were housed at 24.1°C and 30% humidity with a 11:00 A.M.– 11:00 P.M. light-dark cycle. Spontaneously breathing rats were initially anesthetized by isoflurane (1–2%) in an oxygen-air mixture delivered through a face mask. Then, after being weighed, they were sacrificed by exsanguination from a cardiotomy performed under deep isoflurane anesthesia. During exsanguination, the head was elevated to ensure elimination of blood from the brain. The whole brain, including olfactory bulbs, cerebellum, and brainstem, was procured, weighed, dried at 35–38°C for 2 days, and weighed again. Brain water content was calculated as follows: % H_2_O = (1 –dry weight/wet weight) x 100 [11]. A total of 129 animals were studied in 12 groups. Each group was of a different average age and weight when ordered from the supplier, ranged in number from 2 to 20 animals, and did not overlap in age with other groups. The median [interquartile range (IQR)] group size was 9 [8, 12] rats.

### Statistical analysis

All data are presented as median [IQR], and linear and nonlinear regression was used to establish continuous relationships between brain water content, age, and weight. We checked the linear regression to determine if additional terms would contribute to the quality of the fit. The mean and standard error of the mean (SE) are reported for each regression parameter, along with the overall quality of the fit (R^2^). We initially chose the nonlinear functions for data fitting more for convenience than to validate any preconceived theoretical concept regarding the nature of these relationships but, as detailed in the Discussion, they relate to classical concepts of allometric scaling [12]. Analysis was facilitated with Mathematica 12.1 (Wolfram Research, Inc., Champaign, IL, U.S.A.).

## Results

**Fig 1:**
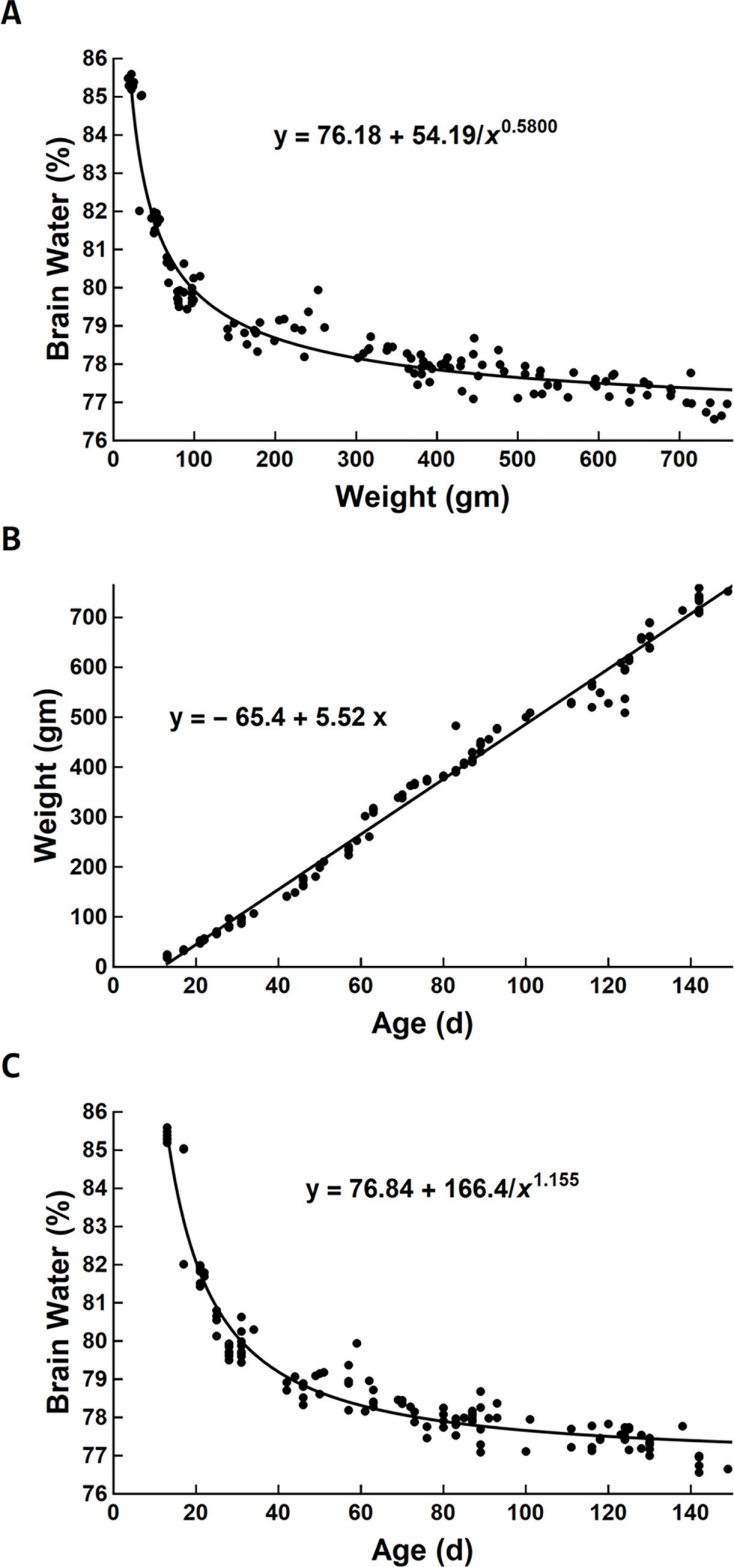
Curve fitting revealed a nonlinear relationship between brain water content and weight (A), a linear relationship between weight and age (B), and a nonlinear relationship between brain water content and age (C) for all 129 rats from the study. The corresponding regression equations are given in each panel, where the functional forms of the nonlinear relationships in A and C are the same but with different parameters. A quadratic term for the linear regression in panel B was not contributory (*p* = 0.30). All parameters of the regression equations in each panel are significantly different from zero (*p* < 0.001) and are given along with their standard errors and quality of fit (R^2^) in Table 1.

Overall, the 129 male Sprague-Dawley rats ranged in age (weight) from 13 to 149 days (18 to 759 g). Brain water content decreased from 85.59% to 76.56% with increasing age (weight). Results from the entire study population are shown in Fig 1 and available in the S1 Data. The regression equations are shown on each figure panel, and additional detail regarding curves to which the data were fit are provided in Table 1. Fig 1A illustrates a monotonic curvilinear decrease in brain water content with weight that begins to flatten for the largest animals. The variation observed in brain water between the extremes of weight was almost 10%. Because weight and age for the study animals was linearly related over the entire study population (Fig 1B), brain water as a function of age in Fig 1C was fit with the same function as that used in Fig 1A.

**Table 1:** Details of parameters for regression equations displayed in panels of Fig 1. All parameters are significantly different from zero (*p* < 0.001).

Regression equation	Parameter 1	Parameter 2	Parameter 3	R^2^
	mean (SE)	mean (SE)	mean (SE)	
Fig 1, panel A	76.18 (0.26)	54.19 (1.18)	0.5800 (0.0431)	>0.99
Fig 1, panel B	-65.4 (4.2)	5.52 (0.05)	NA	0.99
Fig 1, panel C	76.84 (0.16)	166.42 (1.61)	1.155 (0.066)	>0.99

NA = not applicable; SE, standard error of the mean.

## Discussion

For rats ranging in age (weight) from 13 to 149 days (18 to 759 g), brain water content declined by almost 10% of total brain weight. This decrease should be compared to those in studies of osmotherapy intended to determine the reduction in brain water associated with such therapy, where typical changes in brain water are on the order of 1.5% [2, 3]. This finding emphasizes the importance of using control animals of identical age in experiments involving measurement of brain water, particularly when studying young animals, in which the brain water-age curve is particularly steep. The magnitude of the decrease in brain water content with age and its curvilinear shape with a steep initial descent is similar to what has been observed in humans and rabbits by MRI [4, 5]. The data from MRI reflect progressive myelination with development [6–8] and are also consistent with the age-dependent expression of aquaporin channels in the brain [13].

When interpreting the results of our study, it is important to recognize that we examined rats only up to 149 days of age. This duration is only a fraction of the lifespan of the Sprague-Dawley rat, which can live to over 3 years and weigh on the order of 500–700 g [14, 15]. Although the younger end of the age range of 13 days is somewhat greater than zero, this starting point approximates the level of brain development of a newborn human [15]. Furthermore, by postnatal day 20–21, the rat brain reaches 90–95% of its adult weight, attains its peak myelination rate, and exhibits >50% of adult-level synaptic density [16]. Clearly, rats may live for several years longer than those of the current study. To extend the study over the animal’s full age range while sampling brain water content to the same extent as in this study would require an order of magnitude more animals, most housed for one or more years. Such a study is somewhat impractical and would require a design different from this one to provide similar data for the older portion of the age spectrum. Samples would need to be collected at more discrete points along the age spectrum, as has been done for MRI studies of the aging brain. These MRI studies have revealed both anatomic and mechanistic heterogeneity underlying differences in atrophy, myelin, and free water reduction with age [9, 10]. Our study extends only to early adulthood, to the boundary of where age and weight remain proportional. Up until this point, either age or weight could be used to control for expected levels of brain water. Beyond this, age is likely to be more important.

The nonlinear equations used to fit the data were initially chosen empirically for their utility in doing just that. However, we retained them, not only for their ability to fit the data but because the nonlinear term of the form ax^b^ in the equation used to fit the data, where a and b are coefficients and x is the independent variable, is frequently used in allometric models [12]. The clear linear relationship between weight and age for the available data permitted brain water content to be related to each of these variables using equations of the same functional form. A much smaller study that used Wistar rats over approximately the same age range as those in our study [17] reported an inflection point in the weight-age curve that was clearly not observed here. Whether the lack of a similar inflection point in our somewhat larger study represents differences in how the animals were fed and maintained, differences in the type of rat (Wistar vs. Sprague-Dawley), or something more fundamental is not apparent.

One additional limitation of our study was the use of only male rats. This single-sex study is consistent with prior studies of osmotherapy with mannitol [2, 3], in which researchers used only male animals to avoid any possible confounding from the established vascular effects of estrogen, particularly altered membrane permeability [18]. These prior observations along with those of the current study will facilitate the conduct of future studies with both sexes. Importantly, studies of female rats will need to address both the onset of puberty and any variations during the estrous cycle. Because puberty occurs at approximately 7 weeks of age in Sprague-Dawley rats and this point lies in a region of moderate change in brain water content with age (Fig 1), any hormonal variation in brain water content not controlled for could obscure age-related effects.

## Conclusions

In normal male Sprague-Dawley rats, brain water content expressed as a percentage of total brain weight declines in a steep curvilinear fashion by almost 10% over the early phase of life and asymptotes in early adulthood.

## Supporting information

S1 Data Original data used to generate Fig 1. The three columns of data for 129 animals indicate the weight (grams), brain water content (%), and age (days) of each animal.
(XLSX)
